# Notes from the Cell Cycle World: Less Is More

**DOI:** 10.1371/journal.pbio.1001748

**Published:** 2013-12-31

**Authors:** Mary Hoff

**Affiliations:** Freelance Science Writer, Stillwater, Minnesota, United States of America

[Fig pbio-1001748-g001]What does it take to turn one bacterial cell into two very different ones? Not nearly as much as we thought, according to a new study by Martin Howard, Patrick Viollier, Seán M. Murray, Gaël Panis, and colleagues.

**Figure pbio-1001748-g001:**
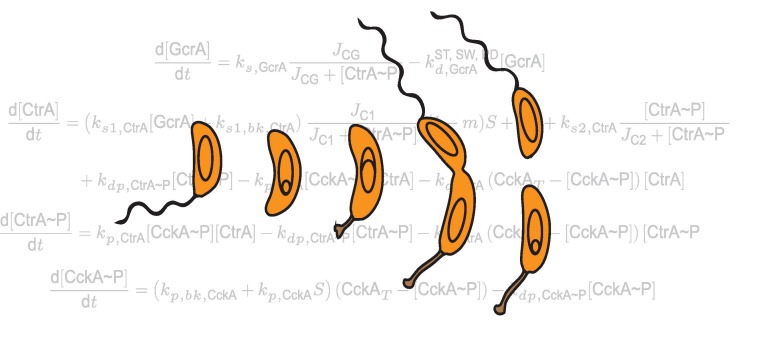
Minimal mathematical modeling and forward genetics reveals that an asymmetric cell cycle in *Caulobacter crescentus* can be controlled by a simpler regulatory network than previously thought.

*Caulobacter crescentus*, a bacterium ubiquitous in water, forms two distinct body types. The first, a version with a stalk that sticks to surfaces, is capable of going through the cell cycle, which makes two cells from one. The other, “swarmer” cell, which has a tail and swims, cannot reproduce until it morphs into the stationary type. This mix of simple (one cell dividing) plus complex (two distinct products) has made *C. crescentus* a model organism for studying the cell division cycle.

Studies so far have discovered that four key proteins regulate the process: DnaA, GcrA, CtrA, CcrM. These four proteins are sequentially produced and destroyed as the cell goes through the process of replicating its DNA, enlarging its membrane and cell wall, and creating an internal cell environment that allows the offspring cell to form a tail rather than a stalk. Their jobs are those of the gears within an old-fashioned clock: to keep things happening in the right order and for the right amount of time.

But are all these proteins absolutely necessary for replication to take place, or might some be the frosting on the cake, fine-tuning the process and products but not really indispensable for cell cycling?

To test that, Howard and colleagues developed a simple model, based largely on data reported in previous studies, that mathematically describes: (1) changes over time and location in the activity of GcrA, which regulates the process of dividing the cell; (2) changes over time and location in the activity of CtrA and another protein known as CckA, which regulates CtrA and orchestrates the processes that determine whether the daughter cell is stalked, stationary, and able to reproduce or tailed, mobile, and unable to reproduce; and (3) three key cell cycle events: differentiation, replication, and compartmentalization into the two types of daughter cells. To their surprise, the model predicted—contrary to previous experimental observation—that GcrA is not needed for *C. crescentus* to successfully undergo replication into the two different daughter cells.

How could that be, given that others had previously observed that mutants lacking the ability to make GcrA were unable to reproduce? The team decided to find out by inactivating the gene that makes GcrA. They discovered that cells without functioning GcrA could indeed reproduce, though colony formation took three times longer than normal, which may be why previous observers concluded they cannot reproduce. However, they also noted that daughter cells had defects in chromosome number due to increased cell length, a decreased ability to float and swim, and changes in the abundance of the other regulatory proteins. Further studying the daughter cells, the researchers concluded that the defects were due to reduced levels of a cell division protein, FtsN, that GcrA regulates.

In the process of experimentally verifying their modeled results, the researchers encountered yet another intriguing discovery: they found that the methylase CcrM is also not required for successful cell cycling, although, as was the case for GcrA, its absence slows the process. Intriguingly, they also found that when CcrM was absent in cells that also lacked the ability to make GcrA, those cells actually reproduced with less severe defects than did cells missing GcrA alone, although the lengthened time to reproduce was not substantially affected. Again, FtsN was among the proteins implicated in deficiencies in the experimentally altered cells. Returning to the mathematical model, the researchers were able to show how the model could explain the lack of real recovery in doubling time when both CcrM and GcrA are absent, adding further support for the model's robustness.

Checking genetic records of other, related bacterial species, the researchers found that the genes coding for the two “dispensable” proteins are consistently either both present or both absent. They concluded that GcrA/CcrM functions as an independent genetic module that, though it contributes robustness to the cell cycle process, is not a sine qua non for its completion.

The research team noted that many aspects of the *C. crescentus* cell cycle will need to be elucidated before we have a clear picture of what's going on inside these apparently simple organisms when the time comes to reproduce. Nevertheless, the current study not only sheds valuable light on the most basic aspects of asymmetric cell cycle control, showing them to be even more basic than previously thought, but also provides a valuable mathematical modeling tool for defining and exploring the fundamental regulatory mechanisms behind other, more complex cell cycles, including those of eukaryotes.

**Murray SM, Panis G, Fumeaux C, Viollier PH, Howard M (2013) Computational and Genetic Reduction of a Cell Cycle to Its Simplest, Primordial Components** doi:10.1371/journal.pbio.1001749

